# Risk factors for recurrent wheezing after bronchiolitis

**DOI:** 10.1186/s12887-023-04108-9

**Published:** 2023-06-23

**Authors:** YH Fan, PL Zhang, YJ Huang, C Xie, T Ai

**Affiliations:** Department of Pediatric Pulmonary Chengdu Women’s and Children’s Central Hospital, School of Medicine, University of Electronic Science and Technology of China, Chengdu, 611731 China

**Keywords:** Recurrent wheezing, Bronchiolitis, Infant

## Abstract

**Background:**

This study aimed to determine whether there was an association between certain factors in patients with bronchiolitis and recurrent wheezing in childhood.

**Method:**

In 2021 we tracked children hospitalized for bronchiolitis at Chengdu Women’s and Children’s Central Hospital in 2017. The patients were classified into recurrent wheezing group (RWG) and non-recurrent wheezing group (NRWG). Possible risk factors including maternal age, school-age siblings, allergic history, atopic dermatitis, allergic rhinitis, atopic family history, severity of the condition, duration of hospitalization, nasopharyngeal secretions culture, blood eosinophil counts, FeNO and skin prick test were compared between the two groups. Continuous variables were analyzed by independent sample t-test for normal distribution and Mann-Whitney U-test for non-normal distribution. Categorical variables were tested using chi-square tests. Multifactor analysis was conducted by stepwise logistics regression analysis.

**Results:**

In total 167 participants were included, of which 26 and 141 were in RWG and NRWG respectively. In RWG children represented higher maternal age (P = 0.02) and greater probability of allergic history, atopic dermatitis, allergic rhinitis, atopic family history (odds ratio [OR] = 4.0,3.7, 7.8, 10.9 respectively, P < 0.01). However, school-age siblings, severity of the condition, duration of hospitalization, blood eosinophil counts, fractional exhaled nitric oxide and skin prick test results seemed unrelated to recurrent wheezing. In the subgroup analysis of nasopharyngeal secretion culture, there were more Moraxella catarrhalis-positive in RWG(P = 0.043). Atopic dermatitis, allergic rhinitis and atopic family history were identified as independent risk factors for recurrent wheezing.

**Conclusion:**

Some children with bronchiolitis will develop recurrent wheezing, and the risk factors are allergic history, Moraxella catarrhalis infection or colonization, atopic dermatitis, allergic rhinitis and atopic family history; the latter three are independent risk factors.

## Introduction

Bronchiolitis is one of the most common lower respiratory tract infections in infants and children under 2years of age. The most common cause is viral infection particularly respiratory syncytial virus(RSV)infection. The main clinical manifestations are rhinitis, cough, paroxysmal wheezing, tachypnea, rales and the use of accessory muscles. In addition, children often develop acute inflammation, mucosal edema, epithelial cell necrosis, mucus secretion, bronchiolar stenosis and obstruction. Furthermore, viral infection often causes airway hyperreactivity in children, which is closely related to the occurrence of recurrent wheezing and asthma [1].

Carroll et al. reported a relationship between the increasing severity of bronchiolitis and higher odds of developing childhood asthma [2]. Some cohort studies have estimated that 30–40%of infants with severe bronchiolitis develop childhood asthma [3]. This study aimed to determine whether there was an association between some factors in patients with bronchiolitis and recurrent wheezing in childhood.

## Method and materials

### Study design and participants

A retrospective study was conducted by searching the electronic medical record system in 2017 of Chengdu Women’s and Children’s Central Hospital patients diagnosed with bronchiolitis in 2017. The diagnostic criteria for bronchiolitis were based on the clinical practice guidelines of the American Academy of Pediatrics [1]. The exclusion criteria included: 1) premature and 2) patients with other diseases such as congenital heart disease, chronic lung disease. In January 2021, the included patients’ guardians were contacted by phone and asked to complete a questionnaire about the number of times their children had wheezed in the past 4 years after bronchiolitis. In addition, other information such as general information history of allergy, family history, presence of siblings and maternal age were collected. Furthermore the patients were classified into the recurrent wheezing group (RWG) and non-recurrent wheezing group (NRWG) and possible risk factors were compared between the two groups.

### Definition of recurrent wheezing and severity scores

Patients with recurrent wheezing experienced two or more wheezing episodes following the initial episode of bronchiolitis. The severity scores referred to as Wang’s clinical score system [1] including the level of respiratory rates, wheezing, three depression signs and general situation.

### Measurement of blood eosinophils

The hospital laboratory measured blood eosinophil counts during the hospitalization with a Automated hematology analyzer XNseries by SYSMEX CORPORATION. These blood samples were obtained from the patients in the first 24 h after admission.

### Detection of respiratory bacteria

A nurse aspirated the patients’ nasopharyngeal secretions and placed them in a sterile tube on the first day of their hospitalization for bronchiolitis and respiratory bacteria were detected by nasopharyngeal secretion culture.

### Detection of allergen and fractional exhaled nitric oxide(FeNO)

The detection of allergens was based on the skin prick test using reagents such as dust mites, cockroaches, pollen, eggs and milk. FeNO was detected by a nitric oxide detector produced by Guangzhou Purui Medical Technology Co. LTD (N1, 20200302) using an electrochemical method. Tide assay was used in all patients owing to their young age.

### Statistical analysis

Data were analyzed using SPSS Statistics 26. Continuous variables were expressed as mean ± standard deviation using an independent sample t-test for normal distribution and median (lower quartiles, upper quartiles) using Mann-Whitney U-test for non-normal distribution. Categorical variables were tested using chi-square tests or Fisher’s exact test and statistical significance was set at P < 0.05. Multifactor analysis was conducted by logistics regression analysis. Stepwise regression was used including all variables.

## Results

Out of 181 patients included in the research, 167 were successfully contacted with 26 patients classified in the RWG and 141 in the NRWG.

### Demographic and clinical characteristics of analyzed patients

There were no significant differences between the RWG and NRWG for age at hospitalization, sex, severity score or duration of hospitalization (Table 1).

**Table 1:** Demographic and clinical characteristics of recurrent wheezing group (RWG) and non-recurrent wheezing group (NRWG)

	RWG	NRWG	P
Age at enrollment, months, mean (SD)	8.2(3.2)	7.8(4.3)	0.71
Gender, boys, n (%)	15(57.7)	103(73.0)	0.114
Maternal age, years, (p25, p75)	28(27, 30.25)	28(26, 29)	0.02
School-age siblings, n (%)	10(38.5)	37(26.2)	0.203
Allergic history, n (%)	10(38.5)	19(13.5)	0.004
Atopic dermatitis, n (%)	19(73.1)	60(42.6)	0.004
Allergic rhinitis, n (%)	13(50.0)	16(11.3)	< 0.001
Atopic family history, n (%)	18(69.2)	24(17.1)	< 0.001
Severity score, median (p25, p75)^a^	3(2, 3.75)	3(2, 5)	0.781^b^
Duration of hospitalization, days, median (p25, p75)	6(5, 6.75)	6(5, 7)	0.099^b^

SD: Standard Deviation; n: sample size

a: The severity scores referred to the Wang’s clinical score system, which included the level of respiratory rates, wheezing, three depressions sign and general situation

b:Mann-Whitney U-test was used because of the non-normal distributions

In addition, there were no statistically significant differences between the two groups regarding the presence of school-age siblings and the seasons when they were diagnosed. However, for maternal age, RWG showed older age than NRWG (P = 0.02). Further, according to this result, we used ROC curves to look for a cutoff of 26.5 years old. Considering the maternal age < 26.5 years and labor age > 26.5 years, we found that children of mothers aged > 26.5 years when delivering were more prone to recurrent wheezing following bronchiolitis (OR 9.41,95%CI 1.23–71.89, P 0.01). Allergic history, atopic dermatitis, allergic rhinitis and atopic family history showed a higher incidence in RWG than in NRWG (odds ratio[OR]: 4.0, 3.7, 7.8, 10.9, respectively, P < 0.01). Notably only 139 patients in NRWG were considered in the statistical analysis of hospitalization duration and maternal age. This was because two patients requested discharge before they fully recovered and two patients’ guardians forgot to provide their maternal age information.

### The influence of respiratory bacteria and antibiotic use

A nasopharyngeal secretion culture test was performed on 165 patients and 34 samples showed positive results; of these, 8 samples (32.0%) were positive in RWG and 26 (18.6%) in NRWG with no statistical difference between the two groups (P = 0.126). The number of Streptococcus pneumoniae-positive, Staphylococcus aureus-positive, Moraxella catarrhalis-positive and Haemophilus influenzae-positive in RWG were 1, 1, 4, 2, respectively and in NRWG were 5, 2, 6, 13, respectively. After subgroup analysis, the positive rate of Moraxella catarrhalis was significantly higher in RWG than in NRWG (P = 0.043). No statistical differences between the two groups were observed for the other three bacteria (Table 2).

**Table 2:** Nasopharyngeal secretions culture results in recurrent wheezing group (RWG) and non-recurrent wheezing group (NRWG)

	RWG	NRWG	P
Bacteria negative	17	114	
Bacteria positive	8	26	0.126
Streptococcus pneumoniae-positive	1	5	0.578^a^
Staphyloccocus aureus-positive	1	2	0.354^a^
Moraxella catarrhalis-positive	4	6	0.043^a^
Haemophilus influenzae-positive	2	13	1.000^a^

a: Fisher’s exact test was used

When the patients were diagnosed with bronchiolitis, no significant difference was found in the use of antibiotics between the RWG and NRWG (P = 0.267). The two most commonly used antibiotics were β-lactam antibiotics and macrolides which were sometimes used in combination. However there was no significant difference between the two groups in terms of usage of β-lactam antibiotics, macrolides or their combination (Table 3).

**Table 3:** Antibiotics use in recurrent wheezing group (RWG) and non-recurrent wheezing group (NRWG)

	RWG	NRWG	P
Without antibiotics	12	49	
With antibiotics	14	92	0.267
β-lactam antibiotics	9	51	0.498
Macrolides	3	21	0.540^a^
Combinated antibiotics	2	20	0.335^a^

a: Fisher’s exact test was used

### The influence of allergy-related tests

A total of 166 patients completed the blood eosinophil count. There was no significant difference in eosinophil counts or proportions between the RWG and NRWG. The FeNO levels were similar (Table 4), of which 151 patients were included.

**Table 4:** Blood eosinophil counts and FeNO of recurrent wheezing group (RWG) and non-recurrent wheezing group (NRWG)

	RWG	NRWG	P
Blood eosinophil counts, *10^9/L, median (p25, p75)	0.13(0.05, 0.28)	0.07(0.03, 0.22)	0.145^a^
Blood eosinophil proportions, %, median (p25, p75)	0.40(0.01, 2.28)	0.1(0.01, 1.40)	0.136^a^
FeNO, ppb, median (p25, p75)	11.5(9, 14.8)	12(8, 15)	0.856^a^

SD: Standard Deviation; n: sample size; FeNO: fractional exhaled nitric oxide

a: Mann-Whitney U-test was used because of the non-normal distributions

A total of 141 patients underwent the skin prick test. Of these, 9(39.1%) patients in RWG and 41(34.7%) patients in NRWG were positive, which was not significantly different (P = 0.688). Among the different categories of allergens, food allergens were the most common in these two groups. The two groups had no statistical difference in positive inhaled, food or combined allergens (Table 5).

**Table 5:** The results of skin prick test of recurrent wheezing group (RWG) and non-recurrent wheezing group (NRWG)

	RWG	NRWG	P
Skin prick test negative	14	77	
Skin prick test positive	9	41	0.688
Inhaled allergen positive	3	7	0.366^a^
Food allergen positive	4	21	1.000^a^
Combinated allergen positive	2	13	1.000^a^

a: Fisher’s exact test was used

### Analysis of risk factors of recurrent wheezing after bronchiolitis

We included nearly all factors in the model (age at enrollment, gender, maternal age, school-age siblings, allergic history, atopic dermatitis, allergic rhinitis, atopic family history, severity score, duration of hospitalization, Moraxella catarrhalis-positive, blood eosinophil counts and proportions). Finally, we found atopic dermatitis, allergic rhinitis and atopic family history were identified as independent risk factors for recurrent wheezing (Table 6).

**Table 6:** Analysis of risk factors of recurrent wheezing after bronchiolitis by logistic regression analysis

Predictive variable	P	OR	95%CI
Atopic dermatitis	0.049	2.88	1.00-8.28
Allergic rhinitis	0.018	3.76	1.26–11.19
Atopic family history	< 0.001	7.32	2.58–20.77

## Discussion

It is now widely accepted that bronchiolitis is associated with future progression to recurrent wheezing and asthma [4–6]. Over the years, several studies have explored the risk factors associated with recurrent wheezing or asthma in children with bronchiolitis in their early life [7–19]. Some of these studies have suggested that the severity of bronchiolitis is related to recurrent wheezing in future [13, 14, 20, 21]. Be’er [17] reported that children admitted to the pediatric intensive care unit for bronchiolitis were more likely to be associated with asthma in early life.

Furthermore, Chen [15] followed up 81 infants in China for 2 years and found that moderate-severe bronchiolitis were the risk factors for recurrent wheezing. However we did not find a significant relationship between disease severity and future recurrent wheezing in terms of severity score or hospitalization duration. The reason why our conclusions were different may be the inconsistent target outcomes observed in all studies.

In this study, we found that older maternal age (> 26.5 years old) may be associated with recurrent wheezing in children diagnosed with bronchiolitis in early life but was not an independent risk factor. Another study [22] examined the association between maternal age and asthma and concluded that maternal age less than 20 years old was a protective factor. The reason for this is still unclear and further research is needed.

It is known that atopic status is associated with recurrent wheezing. A previous review [23] indicated that atopic status and genetics play a decisive role in recurrent wheezing and asthma. Singla [24] examined 260 children with recurrent wheezing and reported 35% with allergic rhinitis. Chen [15] found that the history of eczema was the only independent risk factor among the factors they selected. Moreover we found that allergic history, atopic dermatitis, allergic rhinitis and atopic family history were all related to recurrent wheezing after bronchiolitis and the latter three were independent risk factors. Therefore this suggests that for infants with allergic history, atopic dermatitis, allergic rhinitis or atopic family history, more active measures should be taken to prevent the occurrence of recurrent wheezing after bronchiolitis. However, according to our research, skin prick test, FeNO and blood eosinophil counts were not significantly associated with recurrent wheezing after bronchiolitis. Pesonen [25] found skin prick test positivity in children aged 5 years could predict recurrent wheezing at the age of 11 and 20 years. In contrast, our study involved a skin prick test for infants/toddlers, and maybe the accuracy of the test needed to be evaluated by following up the results of this test when the children grow up. A cohort study [26] concluded that sedated single-breath FeNO in infants/toddlers could predict asthma at the age of 6 years. However, in another review [27], they identified that FeNO was used only to estimate eosinophil inflammation rather than asthma. Gaillard [28] suggested that blood eosinophil counts in infants with wheezing were a risk factor for persistent wheezing at school age. Wagener [29] found that blood eosinophil counts were highly correlated with those in sputum considered a widely used biomarker for asthma [27]. Midulla [30] reported that blood eosinophil counts were the main risk factors for recurrent wheezing in children diagnosed with bronchiolitis; however it has also been suggested that the alveolar lavage fluid eosinophil counts may have a limited value in predicting recurrent wheezing [31]. Piippo-Savolainen [32] found that blood eosinophil counts during bronchiolitis were not related to recurrent wheezing in adults. In conclusion, more researches should be conducted on the effects of skin prick test, FeNO and blood eosinophil testing during bronchiolitis on recurrent wheezing in the future.

In our analyses, a parallel analysis of the two groups showed that the predominant bacterial species in the nasopharynx were Streptococcus pneumoniae, Staphylococcus aureus, Moraxella catarrhalis and Haemophilus influenzae. Infection or colonization of Moraxella catarrhalis was a risk factor for later recurrent wheezing in children with bronchiolitis. Previous studies on airway microbiota have implicated Moraxella catarrhalis as a risk factor for wheezing and asthma using both cross-sectional and prospective study designs [33–35]. Moraxella catarrhalis was found to be associated with chronic wheezing in children’s later life [33, 34] which was also confirmed in our study.

## Conclusion

A proportion of children with bronchiolitis will develop recurrent wheezing and the risk factors are allergic history, Moraxella catarrhalis infection or colonization, atopic dermatitis, allergic rhinitis, atopic family history, and latter three are independent risk factors.

## Data Availability

The datasets used and/or analyzed during the current study are available from the corresponding author on reasonable request.
